# Dr. William Henry Watson

**Published:** 1913-02

**Authors:** 


### OBITUARIES.

---

Dr. William Henry Watson, Hahnemann, 1884, died at his home in Utica, January 2, aged 83.

